# Ethylene and 1-methylcyclopropene modulate aroma volatile profiles in kiwifruit (*Actinidia* spp.) during postharvest storage

**DOI:** 10.1007/s10068-026-02248-z

**Published:** 2026-09-03

**Authors:** Inhwan Kim, Eunyoung Park, Hyunjun Lee, Donghee Ahn, Jeong Hee Choi, Kee-Jai Park, Jeong-Ho Lim, Jihyun Lee

**Affiliations:** 1 Department of Food Science and Technology, Chung-Ang University, Anseong, 17546 Republic of Korea; 2 Department of Food and Nutrition, Seoul National University, Seoul, 08826 Republic of Korea; 3 The Research Institute of Human Ecology, Seoul National University, Seoul, 08826 Republic of Korea; 4 Smart Manufacturing Research Group, Korea Food Research Institute, Wanju, 55365 Republic of Korea

**Keywords:** Kiwifruits, Ethylene, 1-Methylcyclopropene, Storage, Aroma compounds, rOAV

## Abstract

Kiwifruit is a climacteric fruit, and ethylene-mediated ripening accelerates quality deterioration during storage. In this study, volatile compound composition was profiled during storage for 0–10 d under control, ethylene, and 1-methylcyclopropene (1-MCP) treatments. A total of 27 volatile compounds were identified, comprising nine aldehydes, seven esters, four alcohols, five terpenes, one ketone, and one furanone. Total volatile contents, expressed as internal standard equivalents, increased to 6335 µg/kg in control fruit and 8771 µg/kg in ethylene-treated fruit by day 10, whereas 1-MCP-treated fruit showed a decline to 71.5% of initial levels. Relative odor activity values (rOAV) indicated aldehyde-driven odor activity at early stages across treatments, followed by increased ester odor activity in the control and ethylene-treated fruits. In contrast, esters remained below key odor activity thresholds throughout storage in 1-MCP-treated fruits. These findings improve the understanding of aroma development in kiwifruit during storage in response to ethylene and 1-MCP treatments.

**Supplementary Information:**

The online version contains supplementary material available at https://doi.org/10.1007/s10068-026-02248-z.

## Introduction

Global kiwifruit (*Actinidia* spp.) production has exceeded 4 million tons annually since 2017, reaching approximately 4.4 million tons by 2024 (FAO, 2025). Among the commercially cultivated cultivars, “Hayward” is mainly harvested because of its characteristic flavor and taste (Salazar et al., 2019).

Kiwifruit is a climacteric fruit that produces ethylene as the ripening hormone (McDonald, 1990; Xu et al., 2019). For export and storage purposes, they are usually harvested when they reach physiological maturity, but remain firm and not fully ripened. At this stage, the flavor, sweetness, and aroma are still underdeveloped and require further ripening with ethylene application (Yan et al., 2022).

Volatile compounds play a major role in determining the characteristic aroma of kiwifruits, and their production is regulated during ripening. Ethylene stimulates the biosynthesis of esters, aldehydes, alcohols, and other aroma-relevant volatiles (Kim et al., 2023). In the “Hayward” cultivar, hexanal, hex-2-enal, and methyl butyrate have been identified as the dominant volatiles contributing to aroma (Mota et al., 2012). Post-harvest ethylene regulation may influence aroma development during storage.

1-Methylcyclopropene (1-MCP) is a competitive inhibitor of ethylene receptors and is used to delay ripening of climacteric fruits (Xu et al., 2019). Suppression of ethylene responses by 1-MCP inhibits the accumulation of volatile compounds associated with aroma. Aroma development is significantly reduced following 1-MCP treatment, with marked decreases in key volatiles, such as methyl benzoate (Huan et al., 2020; Wang et al., 2021). Compared with untreated fruits, 1-MCP-treated kiwifruits exhibit lower concentrations of esters and benzenoid compounds, indicating a negative effect on aroma formation and sensory quality.

Previous postharvest studies have documented changes in volatile concentrations during kiwifruit ripening and storage (Huan et al., 2020; Wang et al., 2021), though volatile concentration profiles lack sensory relevance when interpreted without odor threshold information, as odor thresholds vary substantially among volatiles (Grosch, 2001). While volatile profiles have been characterized in kiwifruit cultivars (Xu et al., 2024), and postharvest effects of 1-MCP on kiwifruit volatile production have been reported (Huan et al., 2020; Wang et al., 2021), rOAV-based evaluation of odor-active volatiles under ethylene or 1-MCP treatment remains limited.

This study investigated the effects of ethylene and 1-MCP on the volatile composition of “Hayward” kiwifruit during postharvest ripening. Volatile compounds were quantified using gas chromatography and their aroma contributions were assessed based on relative odor activity values (rOAV). These results contribute to a quantitative understanding of how ethylene-related regulation influences aroma development-associated volatile accumulation.

## Materials and methods

### Chemicals and reagents

Authentic standards of hexanal, hex-2-enal, nonanal, octenal, benzaldehyde, methyl hexanoate, ethyl octanoate, 1-hexanol, α-pinene, and β-pinene were purchased from Sigma-Aldrich (St. Louis, MO, USA). Internal standards for the quantification of volatile compounds (octanal-*d*_*16*_, n-hexyl-*d*_*13*_ alcohol, and ethyl butyrate-*d*_*3*_) were purchased from C/D/N Isotopes (Pointe-Claire, Quebec, Canada). Nanopure water was obtained using a Milli-Q Direct 8 Water Purification System (Merck Millipore, Billerica, MA, USA).

### Kiwi samples and postharvest treatment

Green kiwifruits *(Actinidia deliciosa* ‘Hayward’ cultivar) were purchased from New Zealand and transported under refrigerated conditions to the Korea Food Research Institute (Wanju, Korea). The kiwifruits were divided into three groups (control, 1-MCP-treated, and ethylene-treated). Postharvest treatments were conducted in 60 L sealed acrylic chambers at 20 °C.

For 1-MCP treatment, 1-MCP gas was generated inside the chamber by activating a precursor mixture at 40 °C for 2 h. After gas generation, the chamber was sealed for an additional 20 h, resulting in a final concentration of 1.4 µL/L. Ethylene-treated fruit were exposed to ethylene gas at a final concentration of 1000 µL/L for 22 h, with gas circulation maintained. In both chambers, 200 g of calcium hydroxide was used to absorb CO_2_. Control fruits were stored under the same conditions without gas exposure.

Approximately 16.1 kg of kiwifruit per group were stored at 20 °C for 0, 3, 5, 7, and 10 days for the analysis of odor-active volatile compounds.

### *Determination of odor-active volatile compounds in kiwifruits by HS-SPME-GC*-*MS*

Volatile compounds were analyzed according to the method described by Choi et al. (Choi et al., 2019). Fresh kiwifruits (n = 30) were ground using a blender (BL682 Auto-IQ, Nutri Ninja, MA, USA), and 5 g of kiwifruit puree was transferred into a headspace vial (Gerstel, MD, USA). A saturated NaCl solution (5 mL) was added to the headspace vial. Then, isotopic internal standards (octanal-*d*_*16*_, n-hexyl-*d*_*13*_ alcohol, and ethyl butyrate-*d*_*3*_) were added to headspace vials at a final concentration of 10 ng/g. The headspace vial was sealed using a silicone crimp cap (Gerstel, MD, USA).

After 10 min of equilibration, volatile compounds were adsorbed for 30 min at 40 °C using a 2 cm SPME fiber coated with divinylbenzene/carboxen/polydimethylsiloxane (DVB/CAR/PDMS) (Supelco, PA, USA), which was selected based on a previous fiber optimization study that identified DVB/CAR/PDMS as the most suitable fiber for fruit volatile analysis (Wang et al., 2019), and thermally desorbed for 30 s at 250 °C in the GC injection port. Volatile compounds were analyzed using a gas chromatograph (7890 B, Agilent Technologies, CA, USA) coupled with a mass-selective detector (5977 B, Agilent Technologies), as described previously (Choi et al., 2019). The desorbed analytes were injected in splitless mode and separated on a DB-WAX capillary column (30 m × 0.25 mm i.d., 0.25 µm film thickness; Agilent Technologies). The oven temperature was held at 40 °C for 2 min, heated to 120 °C at 4 °C/min, held at 120 °C for 5 min, raised to 250 °C at 20 °C/min, and held at 250 °C for 5 min. Helium gas (99.999%) was used as carrier gas and flow rate was 1 mL/min. The mass-selective detector was operated in electron ionization (EI) mode (70 eV). The transfer line, ion source, and quadrupole temperatures were 240 °C, 230 °C, and 150 °C, respectively. The mass spectra of the volatile compounds were recorded from *m/z* 30 to 350 at a rate of 3.06 scans/s.

Volatile compounds were identified by comparing their mass spectra and retention times with those of authentic standards. Volatile compounds without authentic standards were tentatively identified by comparing their mass spectra with the Wiley 9 and NIST 08 mass spectral libraries using a match score cutoff of 80%, and by comparing the Kovats retention index with values reported in the NIST Chemistry WebBook.

The relative concentrations of volatile compounds were calculated as previously described (Choi et al., 2019; Lee et al., 2014). The peak area of the quantifier ion for each compound was divided by the peak area of the quantifier ion for internal standard (I.S). Octanal-d16 was used for the quantification of aldehydes and ketones, n-hexyl-d13 alcohol was used for alcohols, alkanes, and olefins, and ethyl butyrate-d3 was used for esters. Volatile compounds not belonging to these classes were quantified using the internal standard with the closest retention time. The area ratio was converted to relative concentration by multiplying by 10 ng of internal standard per gram of kiwifruit, as shown in the following equation:

$${\mathrm{Concentration}}\,\left( \frac{ng}{g} \right)\, = \,\frac{{{\mathrm{extracted}}\,{\mathrm{ion}}\,{\mathrm{peak}}\,{\mathrm{area}}\,{\mathrm{of}}\,{\mathrm{each}}\,{\mathrm{compound}}}}{{{\mathrm{extracted}}\,{\mathrm{ion}}\,{\mathrm{peak}}\,{\mathrm{area}}\,{\mathrm{of}}\,{\mathrm{I}}.\,{\mathrm{S}}.}}\,\left[ {{\mathrm{I}}.\,{\mathrm{S}}.\,\left( \frac{10ng}{g} \right)} \right]$$

The rOAV was calculated based on the relative concentration of volatiles to evaluate the contribution of individual volatiles to the kiwifruit aroma profile (Su et al., 2022). The OAV was calculated by dividing concentration of each compound to threshold of the compound reported literatures. The OAV of each volatile compound was used to estimate the rOAV of individual volatile compounds. The rOAV of each compound was calculated as the ratio of the highest OAV among the volatile compound. Volatile compounds with rOAV ≥ 1 were classified as critical volatile compounds to characteristic aroma in samples (Su et al., 2022), those with 0.1 < rOAV < 1 as contributors to overall flavor (Yin et al., 2019), and those with rOAV < 0.1 as potential aroma compounds (Zhang et al., 2023).

### Statistical analysis

Statistical analyses were performed using SPSS Statistics 23 (SPSS Inc., Chicago, IL, USA), XLSTAT (ver. 2017.03, Microsoft Excel Add-in software, New York, USA) software, and GraphPad Prism version 10.5 (GraphPad Software, Boston, MA, USA). Relative concentrations were summed within each volatile compound class, and the effects of postharvest treatment, storage period, and their interaction were evaluated by two-way analysis of variance (ANOVA) for each compound class and total volatile content. Individual volatile compounds were analyzed within each treatment group by one-way ANOVA. Day 0 compound class profiles were compared among treatment groups using the Kruskal–Wallis test. Differences were considered significant at *p* < 0.05.

## Results and discussion

### Identification of volatile compounds

Among the volatiles detected in kiwifruit, 27 compounds were identified, including nine aldehydes, seven esters, four alcohols, five terpenes, one ketone, and one furanone (Table 1 and Supplementary Figure S1). Specifically, ten volatile compounds (i.e., hexanal, hex-2-enal, nonanal, octenal, benzaldehyde, methyl hexanoate, ethyl octanoate, 1-hexanol, α-pinene, and β-pinene) were confirmed by comparing them with the mass spectra and retention times of authentic standards.

**Table 1:** Odor-active compounds in kiwifruits identified by headspace solid-phase microextraction coupled with gas chromatography-mass spectrometry (HS-SPME-GC-MS)

Peak no	IUPAC name	Odor description (JECFA, 2026)	Unknown RI	Literature RI	Extract ion	Newly produced compounds during storage
*Aldehydes*						
6^*^	Hexanal	Green, grassy	1088	1078	**44**, 56, 41	
8	2-Methyl-2-pentenal	Green, grassy	1139	1151	**41**, 98, 69	
14^*^	Hex-2-enal	Green, grassy	1221	1216	**41**, 42, 83	
17^*^	Nonanal	Citrus, green lemon	1396	1390	**57**, 41, 43	
19^*^	Octenal	Green, herbal	1429	1436	**41**, 55, 70	
22	2,4-Heptadienal	Fatty, green	1468	1474	**81**, 53, 110	
23	Decanal	Citrus, floral	1502	1502	**43**, 41, 55	
24^*^	Benzaldehyde	Fruity, nutty	1525	1520	**106**, 77, 105	
25	6-Nonenal	Green, vegetative	1532	1469	**55**, 43, 70	
*Esters*						
1	Ethyl acetate	Fruity, sweet	840	856	**43**, 61, 45	New
3	Methyl butyrate	Fruity, apple, sweet, banana, pineapple	984	989	**43**, 74, 71	New
5	Methyl isobutyrate	Fruity, floral, apple, pineapple	1040	1041	**43**, 71, 59	
9^*^	Methyl hexanoate	Fruity, tropical fruit	1180	1176	**74**, 87, 43	New
20^*^	Ethyl octanoate	Fruity, wine, waxy	1440	1441	**88**, 101, 127	New
27	Methyl benzoate	Floral, cananga, wintergreen	1635	1631	**105**, 77, 136	
28	Ethyl benzoate	Fruity, sweet, wintergreen	1676	1672	**105**, 77, 122	New
*Ketones*						
31	β-Damascenone	Floral, woody	1828	1814	**69**, 121, 105	
*Alcohols*						
11	3,4-Hexadien-1-ol	Green, herbal	1200		**83**, 55, 41	
16^*^	1-Hexanol	Green, woody	1369	1363	**56**, 43, 41	New
18	2-Hexen-1-ol	Green, unripe banana	1417	1420	**57**, 41, 82	
21	1-Octen-3-ol	Earthy, mushroom	1453	1456	**57**, 43, 72	
*Terpenes*						
4^*^	α-Pinene	Piney, woody	1030	1026	**93**, 91, 92	
7^*^	β-Pinene	Piney, woody	1105	1108	**93**, 41, 69	
10	α-Limonene	Pine, herbal	1192	1196	**68**, 93, 67	
29	α-Terpineol	Citrus, peely	1700	1700	**59**, 93, 121	
30	Verbenone	Camphor menthol, celery	1716	1723	**107**, 135, 91	
*Furanones*						
26	4-Methoxy-2,5-dimethyl-3(2H)-furanone	Sweet, moldy, caramellic	1599	1602	**142**, 43, 71	New

*10 odor-active compounds (i.e., hexanal, hex-2-enal, nonanal, octenal, benzaldehyde, methyl hexanoate, ethyl octanoate, 1-hexanol, α-pinene, and β-pinene) were identified by comparing their mass spectra and retention times with those of authentic standards

The quantifier ion is indicated in bold, and the remaining ions were used as qualifier ions

Volatile profiles differed among the postharvest treatments during storage. Ethyl acetate, methyl butyrate, methyl hexanoate, ethyl octanoate, ethyl benzoate, 1-hexanol, and 4-methoxy-2,5-dimethyl-3(2H)-furanone were not identified on day 0 (fresh fruit, prior to storage) but were detected during storage in the control and ethylene-treated fruit. Ethyl acetate, methyl butyrate, methyl hexanoate, ethyl octanoate, and ethyl benzoate were not identified in 1-MCP treated samples. In a previous study, the contents of ethyl acetate, methyl butyrate, methyl hexanoate, and methyl benzoate significantly increased during storage (Huan et al., 2020).

Methyl butyrate, hex-2-enal, hexanal, and methyl benzoate are the major volatile compounds in kiwifruit (Garcia et al., 2012). In particular, 2,4-heptadienal is a characteristic volatile compound in *Actinidia deliciosa*, which is consistent with its detection in this study (Zhang et al., 2019).

### Multivariate analysis of volatile profiles during storage

**Fig. 1:**
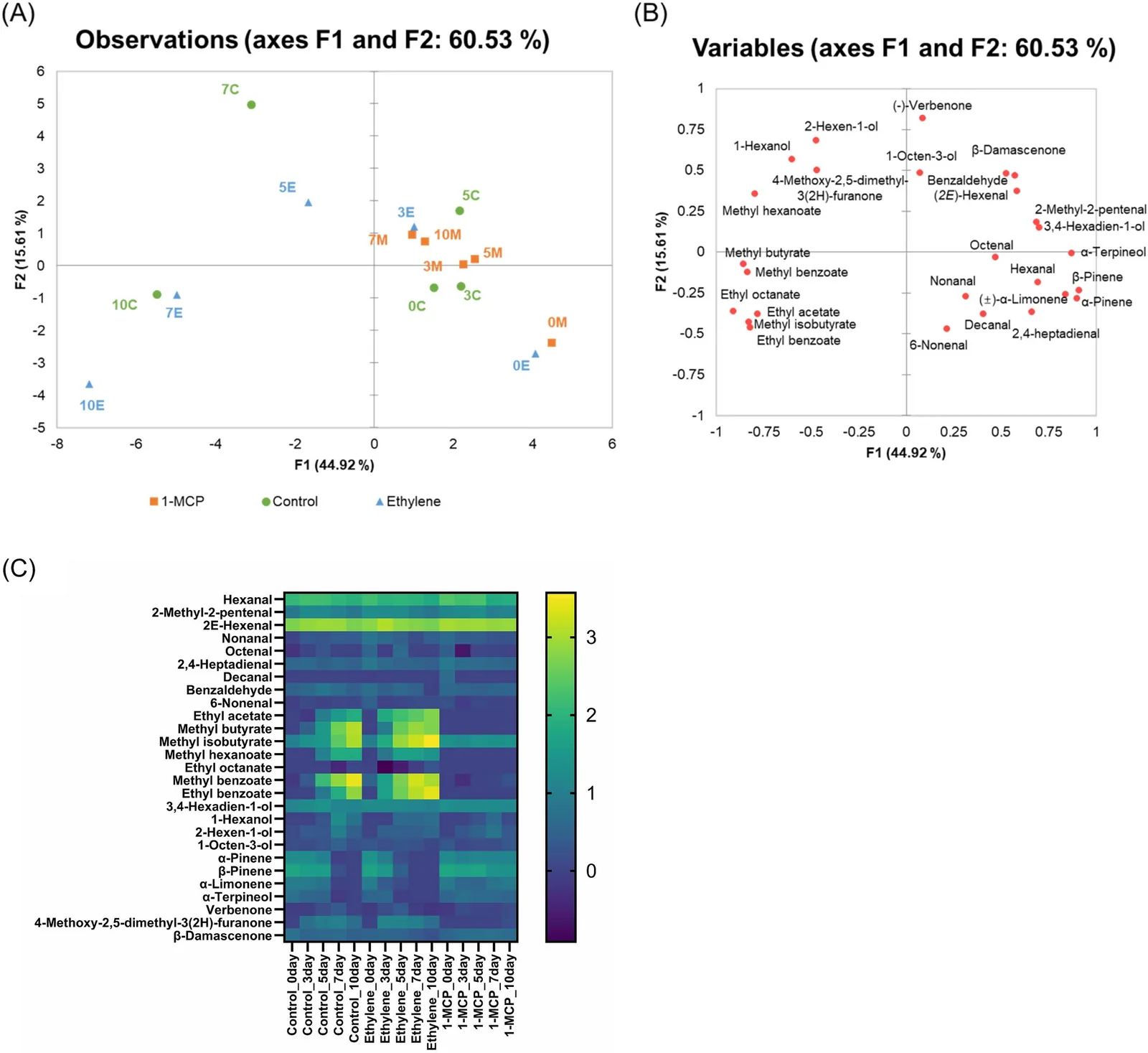
Principal component analysis (PCA) score (**A**), loading plot (**B**), and heatmap visualization (**C**) based on volatile contents of kiwifruits different with postharvest treatment. In the heatmap, the color scale represents the standardized relative abundance of each volatile compound, with colors ranging from purple for lower values to yellow for higher values

PCA and heatmap based on the volatile contents of kiwifruits depending on postharvest treatment and storage are shown in Fig. 1. In the PCA score plot (Fig. 1A), the two principal components explained 60.53% of the total variables, with values of 44.92% and 15.61% for F1 and F2, respectively. The postharvest treated fresh samples (i.e., 0E and 0 M) were separated from the other samples and located on the positive side of F1. In the loading plot (Fig. 1B), the samples were positively correlated with α-pinene. In addition, ethyl benzoate, which is responsible for the fruit and sweet flavor, was spread on the negative side of F1. Among the samples, some of stored samples (i.e., 7C, 10C, 5E, 7E, and 10E) were correlated with ethyl benzoate, suggesting that ethyl benzoate may serve as a potential marker for stored kiwifruits. PCA score plots showed that all 1-MCP-treated samples clustered with early stage control and ethylene-treated samples on the positive side of F1, whereas late-stage control and ethylene-treated samples were separated on the negative side, indicating that 1-MCP treatment maintained an early stage aroma profile throughout storage.

The heatmap based on the volatile content of kiwifruits depending on postharvest treatment and storage are shown in Fig. 1C. Specifically, the stored samples (i.e., 7C, 10C, 5E, 7E, and 10E) had a higher ester content than the other samples.

### Changes in volatile contents during storage

**Fig. 2:**
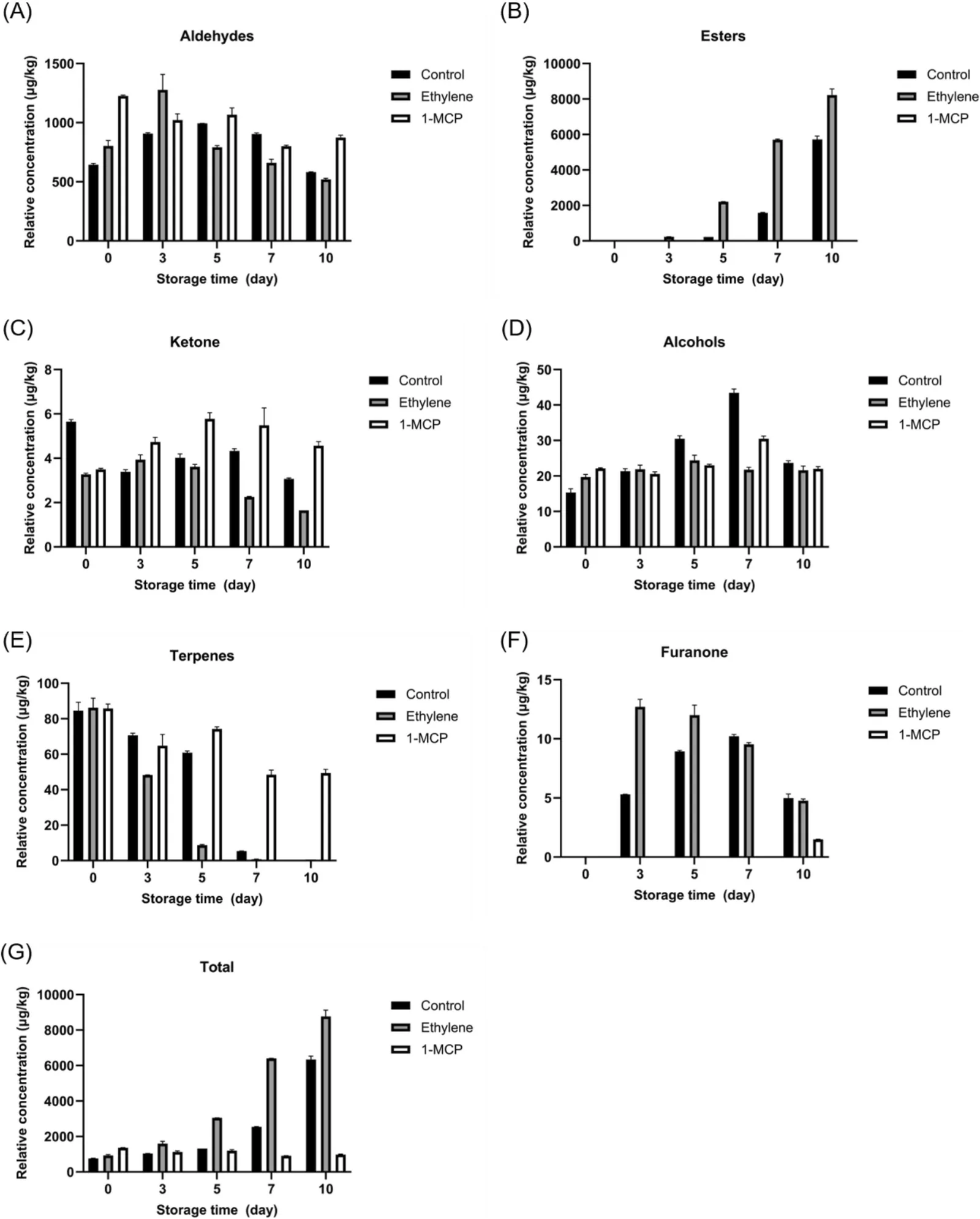
Sum of odor-active compounds in kiwifruits under different postharvest treatments and storage periods. (**A**) Aldehydes, (**B**) esters, (**C**) ketones, (**D**) alcohols, (**E**) terpenes, (**F**) furanone, and (**G**) total volatile content

Storage-dependent changes in volatiles were evaluated by integrating the concentration profiles (Fig. 2) with rOAV-based odor activity (Table 2) under control conditions and following ethylene or 1-MCP application. All volatile concentrations reported herein represent relative concentrations calculated using internal standard equivalents. The sums of relative concentrations for each volatile compound class and total volatile content were significantly affected by postharvest treatment, storage period, and their interaction (*p* < 0.0001). Aldehydes and esters were abundant across conditions and storage periods, and together accounted for more than 80% of the total volatiles. Since aldehydes in kiwifruit are associated with fresh and green notes, whereas esters contribute to fruity and sweet notes, treatment-dependent changes in these compound groups may account for variations in aroma characteristics during storage.

**Table 2:** Odor-active volatile contents (µg/kg, IS equivalents) and relative odor activity values (rOAVs) of kiwifruit under (A) control, (B) ethylene, and (C) 1-MCP treatments during storage at 20 °C for 10 days

(A)											
	Relative concentration (µg/kg)					Threshold^*^ (µg/kg)	rOAV				
	0 day	3 day	5 day	7 day	10 day		0 day	3 day	5 day	7 day	10 day
*Aldehydes*											
Hexanal	115 ± 2 b	167 ± 2 a	158 ± 8 a	106 ± 6 b	87 ± 2 c	2.4	1.70	4.09	3.28	2.04	2.37
2-Methyl-2-pentenal	8 ± 0 c	17 ± 0 a	15 ± 1 a	12 ± 0 b	7 ± 0 c	0.25	1.11	3.95	3.02	2.14	1.91
Hex-2-enal	513 ± 14 d	711 ± 10 c	806 ± 10 a	773 ± 17 b	478 ± 1 e	17	1.07	2.46	2.36	2.10	1.83
Nonanal	n.d	2 ± 0 b	2 ± 0 c	3 ± 0 a	2 ± 0 ab	40	n.d	< 0.01	< 0.01	< 0.01	< 0.01
Octenal	1 ± 0 c	1 ± 0 b	2 ± 0 a	n.d	n.d	0.7	0.03	0.09	0.13	n.d	n.d
2,4-Heptadienal	4 ± 0 b	4 ± 0 a	3 ± 0 c	4 ± 0 ab	3 ± 0 c	56	< 0.01	< 0.01	< 0.01	< 0.01	< 0.01
Benzaldehyde	3 ± 0 d	4 ± 0 c	7 ± 0 a	5 ± 0 b	3 ± 0 d	0.750	0.14	0.30	0.45	0.28	0.25
6-Nonenal	1 ± 0 a	1 ± 0 a	1 ± 0 a	1 ± 0 a	1 ± 0 a	–	–	–	–	–	–
Sum of aldehyde	644 ± 12 c	907 ± 9 b	994 ± 1 a	903 ± 11 b	582 ± 2 d						
*Esters*											
Ethyl acetate	n.d	1 ± 0 d	9 ± 1 c	33 ± 0 b	73 ± 0 a	6200	n.d	< 0.01	< 0.01	< 0.01	< 0.01
Methyl butyrate	n.d	3 ± 0 c	32 ± 1 c	403 ± 16 b	1217 ± 30 a	10	n.d	0.02	0.16	1.86	7.92
Methyl isobutyrate	11 ± 0 c	23 ± 0 c	30 ± 2 c	283 ± 8 b	1067 ± 46 a	7	0.05	0.19	0.22	1.87	9.93
Methyl hexanoate	n.d	n.d	11 ± 0 b	68 ± 1 a	65 ± 4 a	70	n.d	n.d	0.01	0.05	0.06
Ethyl octanoate	n.d	n.d	n.d	n.d	1 ± 0 a	15	n.d	n.d	n.d	n.d	< 0.01
Methyl benzoate	1 ± 0 d	2 ± 0 d	131 ± 1 c	715 ± 5 b	2614 ± 76 a	110	< 0.01	< 0.01	0.06	0.30	1.55
Ethyl benzoate	n.d	n.d	8 ± 1 c	76 ± 1 b	685 ± 32 a	2	n.d	n.d	0.20	1.76	22.30
Sum of esters	11 ± 0 c	29 ± 0 c	222 ± 2 c	1579 ± 27 b	5722 ± 188 a						
*Ketone*											
β-Damascenone	6 ± 0 a	3 ± 0 c	4 ± 0 b	4 ± 0 b	3 ± 0 c	0.002	100.00	100.00	100.00	100.00	100.00
*Alcohols*											
3,4-Hexadien-1-ol	13 ± 1 c	18 ± 1 b	25 ± 0 a	14 ± 0 c	13 ± 0 c	–	–	–	–	–	–
1-Hexanol	n.d	n.d	2 ± 0 c	17 ± 1 a	6 ± 0 b	2500	n.d	n.d	< 0.01	< 0.01	< 0.01
2-Hexen-1-ol	1 ± 0 d	2 ± 0 c	2 ± 0 c	10 ± 0 a	3 ± 0 b	100	< 0.01	< 0.01	< 0.01	< 0.01	< 0.01
1-Octen-3-ol	1 ± 0 bc	1 ± 0 c	2 ± 0 b	3 ± 0 a	2 ± 0 bc	45	< 0.01	< 0.01	< 0.01	< 0.01	< 0.01
Sum of alcohol	15 ± 1 e	21 ± 1 d	30 ± 1 b	43 ± 1 a	24 ± 1 c						
*Terpenes*											
α-Pinene	17 ± 0 b	21 ± 1 a	12 ± 0 c	1 ± 0 d	n.d	190	< 0.01	0.01	< 0.01	< 0.01	n.d
β-Pinene	53 ± 6 a	37 ± 1 b	38 ± 0 b	1 ± 0 c	n.d	140	0.01	0.02	0.01	< 0.01	n.d
α-Limonene	8 ± 0 a	9 ± 0 a	6 ± 0 b	n.d	n.d	60	< 0.01	0.01	0.01	n.d	n.d
α-Terpineol	5 ± 1 a	4 ± 0 b	3 ± 0 b	1 ± 0 b	n.d	0.33	0.58	0.64	0.50	0.10	n.d
Verbenone	1 ± 0 c	1 ± 0 c	1 ± 0 b	2 ± 0 a	n.d	–	–	–	–	–	–
Sum of terpenes	85 ± 5 a	71 ± 1 b	61 ± 1 c	5 ± 0 d	n.d						
*Furanone*											
4-Methoxy-2,5-dimethyl-3(2H)-furanone	n.d	5 ± 0 c	9 ± 0 b	10 ± 0 a	5 ± 0 c	160	n.d	< 0.01	< 0.01	< 0.01	< 0.01
Sum	761 ± 18 e	1036 ± 7 d	1320 ± 2 c	2545 ± 17 b	6335 ± 191 a						

(B)											
	Relative concentration (µg/kg)					Threshold^*^ (µg/kg)	rOAV				
	0 day	3 day	5 day	7 day	10 day		0 day	3 day	5 day	7 day	10 day
*Aldehydes*											
Hexanal	158 ± 7 a	92 ± 4 b	102 ± 2 b	93 ± 2 b	64 ± 2 c	2.4	4.04	1.95	2.36	3.43	2.22
2-Methyl-2-pentenal	18 ± 1 a	17 ± 0 a	17 ± 1 a	10 ± 0 b	7 ± 0 c	0.25	4.36	3.45	3.81	3.51	2.26
Hex-2-enal	602 ± 36 ﻿bc	1154 ± 126 a	657 ± 11 b	549 ± 32 ﻿bc	443 ± 7 c	17	2.17	3.45	2.14	2.87	2.17
Nonanal	7 ± 1 a	4 ± 0 b	3 ± 0 c	1 ± 0 d	3 ± 0 c	40	0.01	0.01	< 0.01	< 0.01	0.01
Octenal	3 ± 0 b	1 ± 0 c	4 ± 0 a	n.d	n.d	0.7	0.30	0.06	0.35	n.d	n.d
2,4-Heptadienal	7 ± 0 a	4 ± 0 b	3 ± 0 cd	3 ± 0 c	3 ± 0 d	56	0.01	< 0.01	< 0.01	0.01	< 0.01
Decanal	1 ± 0 a	n.d	n.d	n.d	n.d	0.003	18.70	n.d	n.d	n.d	n.d
Benzaldehyde	5 ± 1 a	3 ± 0 c	4 ± 0 b	3 ± 0 c	n.d	0.750	0.40	0.21	0.30	0.36	n.d
6-Nonenal	3 ± 0 a	1 ± 0 c	1 ± 0 c	1 ± 0 b	1 ± 0 b	–	–	–	–	–	–
Sum of aldehyde	804 ± 46 b	1277 ± 131 a	792 ± 14 b	661 ± 30 bc﻿	520 ± 9 c						
*Esters*											
Ethyl acetate	n.d	65 ± 2 d	166 ± 8 c	269 ± 0 b	534 ± 25 a	6200	n.d	< 0.01	< 0.01	< 0.01	0.01
Methyl butyrate	n.d	9 ± 0 d	358 ± 9 c	749 ± 30 a	510 ± 32 b	10	n.d	0.04	1.98	6.64	4.24
Methyl isobutyrate	10 ± 0 d	66 ± 1 d	769 ± 32 c	1587 ± 52 b	3709 ± 175 a	7	0.09	0.48	6.07	20.11	44.13
Methyl hexanoate	n.d	7 ± 0 c	40 ± 1 a	39 ± 4 a	27 ± 1 b	70	n.d	0.01	0.03	0.05	0.03
Ethyl octanoate	n.d	n.d	n.d	1 ± 0 b	2 ± 0 a	15	n.d	n.d	n.d	0.01	0.01
Methyl benzoate	3 ± 0 e	43 ± 1 d	486 ± 0 c	1833 ± 4 a	1040 ± 24 b	110	< 0.01	0.02	0.24	1.48	0.79
Ethyl benzoate	n.d	40 ± 3 d	390 ± 0 c	1230 ± 43 b	2401 ± 94 a	2	n.d	1.02	10.77	54.57	100.00
Sum of esters	13 ± 0 d	231 ± 5 d	2209 ± 14 c	5708 ± 38 b	8222 ± 350 a						
*Ketone*											
β-Damascenone	3 ± 0 c	4 ± 0 a	4 ± 0 b	2 ± 0 d	2 ± 0 e	0.002	100.00	100.00	100.00	100.00	68.81
*Alcohols*											
3,4-Hexadien-1-ol	18 ± 1 a	17 ± 0 a	15 ± 1 b	14 ± 0 c	12 ± 0 d	–	–	–	–	–	–
1-Hexanol	n.d	n.d	4 ± 0 ab	4 ± 0 b	5 ± 1 a	2500	n.d	n.d	< 0.01	< 0.01	< 0.01
2-Hexen-1-ol	n.d	3 ± 1 a	3 ± 0 a	3 ± 0 a	4 ± 0 a	100	n.d	< 0.01	< 0.01	< 0.01	< 0.01
1-Octen-3-ol	2 ± 0 a	1 ± 0 b	2 ± 0 a	2 ± 0 b	1 ± 0 c	45	< 0.01	< 0.01	< 0.01	< 0.01	< 0.01
Sum of alcohol	20 ± 1 b	22 ± 1 ab	24 ± 1 a	22 ± 1 ab	22 ± 1 ab						
*Terpenes*											
α-Pinene	24 ± 2 a	10 ± 0 b	1 ± 0 c	n.d	n.d	190	0.01	< 0.01	< 0.01	n.d	n.d
β-Pinene	48 ± 3 a	31 ± 1 b	3 ± 0 c	n.d	n.d	140	0.02	0.01	< 0.01	n.d	n.d
α-Limonene	10 ± 1 a	2 ± 0 b	1 ± 0 c	n.d	n.d	60	0.01	< 0.01	< 0.01	n.d	n.d
α-Terpineol	3 ± 0 a	4 ± 0 a	1 ± 0 b	n.d	n.d	0.33	0.64	0.57	0.14	n.d	n.d
Verbenone	1 ± 0 c	1 ± 0 b	2 ± 0 a	1 ± 0 c	1 ± 0 c	–	–	–	–	–	–
Sum of terpenes	86 ± 5 a	48 ± 0 b	9 ± 0 c	1 ± 0 d	1 ± 0 d						
*Furanone*											
4-Methoxy-2,5-dimethyl-3(2H)-furanone	n.d	13 ± 1 a	12 ± 1 a	10 ± 0 b	5 ± 0 c	160	n.d	< 0.01	< 0.01	0.01	< 0.01
Sum	927 ± 53 e	1594 ± 138 d	3051 ± 3 c	6403 ± 9 b	8771 ± 358 a						

(C)											
	Relative concentration (µg/kg)					Threshold^*^ (µg/kg)	rOAV				
	0 day	3 day	5 day	7 day	10 day		0 day	3 day	5 day	7 day	10 day
*Aldehydes*											
Hexanal	229 ± 15 a	167 ± 16 b	188 ± 10 b	63 ± 3 c	77 ± 9 c	2.4	5.45	2.95	2.72	0.96	1.41
2-Methyl-2-pentenal	14 ± 0 c	18 ± 1 b	20 ± 1 a	10 ± 0 e	13 ± 1 d	0.25	3.31	2.99	2.79	1.52	2.26
Hex-2-enal	963 ± 22 a	826 ± 34 b	847 ± 47 b	716 ± 7 c	772 ± 12 ﻿bc	17	3.24	2.05	1.72	1.54	1.99
Nonanal	4 ± 0 a	2 ± 0 b	2 ± 0 b	2 ± 0 c	2 ± 0 c	40	0.01	< 0.01	< 0.01	< 0.01	< 0.01
Octenal	3 ± 0 a	n.d	1 ± 0 ﻿bc	1 ± 0 b	1 ± 0 c	0.7	0.21	n.d	0.06	0.06	0.06
2,4-Heptadienal	8 ± 0 a	5 ± 0 b	4 ± 0 ﻿bc	4 ± 0 cd	3 ± 0 d	56	0.01	< 0.01	< 0.01	< 0.01	< 0.01
Decanal	4 ± 1 a	n.d	n.d	n.d	n.d	0.003	83.75	n.d	n.d	n.d	n.d
Benzaldehyde	4 ± 0 a	3 ± 0 ﻿bc	3 ± 0 c	3 ± 0 ﻿bc	4 ± 0 ab	0.750	0.29	0.18	0.13	0.16	0.21
6-Nonenal	2 ± 0 a	1 ± 0 d	1 ± 0 b	1 ± 0 ﻿bc	1 ± 0 c	–	–	–	–	–	–
Sum of aldehyde	1231 ± 6 a	1022 ± 52 b	1067 ± 57 b	800 ± 10 c	873 ± 22 c						
*Esters*											
Methyl isobutyrate	23 ± 1 b	18 ± 1 c	26 ± 1 a	24 ± 0 b	24 ± 1 ab	7	0.18	0.11	0.13	0.12	0.15
Methyl benzoate	1 ± 0 a	1 ± 0 a	1 ± 0 a	1 ± 0 a	2 ± 0 a	110	< 0.01	< 0.01	< 0.01	< 0.01	< 0.01
Sum of esters	23 ± 1 c	18 ± 1 d	27 ± 1 a	25 ± 0 ﻿bc	26 ± 1 ab						
*Ketone*											
β-Damascenone	3 ± 0 c	5 ± 0 b	6 ± 0 a	5 ± 1 ab	5 ± 0 b	0.002	100.00	100.00	100.00	100.00	100.00
*Alcohols*											
3,4-Hexadien-1-ol	20 ± 0 a	17 ± 0 c	17 ± 0 d	19 ± 0 b	16 ± 0 d	–	–	–	–	–	–
1-Hexanol	n.d	n.d	2 ± 0 b	4 ± 0 a	1 ± 0 c	2500	n.d	n.d	< 0.01	< 0.01	< 0.01
2-Hexen-1-ol	n.d	2 ± 0 c	3 ± 0 b	6 ± 0 a	2 ± 0 c	100	n.d	< 0.01	< 0.01	< 0.01	< 0.01
1-Octen-3-ol	2 ± 0 a	1 ± 0 b	1 ± 0 b	1 ± 0 b	2 ± 0 a	45	< 0.01	< 0.01	< 0.01	< 0.01	< 0.01
Sum of alcohol	22 ± 0 b	21 ± 1 c	23 ± 0 b	30 ± 1 a	22 ± 1 b						
*Terpenes*											
α-Pinene	18 ± 2 a	12 ± 1 c	14 ± 0 b	13 ± 0 ﻿bc	13 ± 1 ﻿bc	190	0.01	< 0.01	< 0.01	< 0.01	< 0.01
β-Pinene	55 ± 3 a	43 ± 4 b	51 ± 2 a	24 ± 2 c	23 ± 1 c	140	0.02	0.01	0.01	0.01	0.01
α-Limonene	9 ± 1 a	4 ± 0 c	4 ± 0 c	6 ± 0 b	9 ± 0 a	60	0.01	< 0.01	< 0.01	< 0.01	0.01
α-Terpineol	3 ± 0 c	5 ± 0 a	4 ± 0 b	4 ± 0 ﻿bc	4 ± 0 c	0.33	0.60	0.65	0.45	0.44	0.48
Verbenone	1 ± 0 a	1 ± 0 a	1 ± 0 a	1 ± 0 a	1 ± 0 a	–	–	–	–	–	–
Sum of terpenes	86 ± 2 a	65 ± 6 c	74 ± 1 b	48 ± 3 d	49 ± 2 d						
*Furanone*											
4-Methoxy-2,5-dimethyl-3(2H)-furanone	n.d	n.d	n.d	n.d	1 ± 0 a	160	n.d	n.d	n.d	n.d	< 0.01
Sum	1366 ± 10 a	1130 ± 58 b	1197 ± 56 b	909 ± 15 c	976 ± 26 c						

*Threshold values were obtained from literature (Cuevas-Glory et al., 2020; Liu et al., 2022; Noguerol-Pato et al., 2012; Pang et al., 2012; Pino, 2012; Xiao et al., 2017, 2021; Zhai and Granvogl, 2019; Zhang et al., 2021b; Zhao et al., 2023; Zhu et al., 2018)

**Data are reported as mean ± SD. Within each row, values followed by different lowercase letters indicate significant differences between storage times within the same treatment (*p* < 0.05). *n.d.* not detected.

Total volatile content changed differently across treatments during storage, increasing in the control and ethylene groups but decreasing in the 1-MCP samples by day 10. At day 0, the sum of the volatile contents (1366 µg/kg) in 1-MCP samples was 1.47–1.80-fold higher than those in the control (761 µg/kg) and ethylene samples (927 µg/kg). However, a class level comparison of Day 0 volatile profiles showed no significant difference among treatment groups (Kruskal–Wallis test, *p* = 0.8963), indicating that the higher initial total volatile content in the 1-MCP group did not represent a significant difference in the overall compound class profile. During storage, the sum of volatile contents increased to 6335 µg/kg in the control group (8.32-fold relative to day 0) and to 8771 µg/kg in the ethylene group (9.46-fold relative to day 0). In contrast, after 10 days of storage, sum of volatiles in 1-MCP treated kiwifruits was significantly decreased to 71.5% compared to that in 1-MCP treated fresh kiwifruits (1366 µg/kg) (*p* < 0.05).

Aldehyde concentrations decreased during storage across treatments, and Table 2 indicates that rOAV > 1 for key aldehydes (hexanal, 2-methyl-2-pentenal, and hex-2-enal) during storage. In control kiwifruits, hexanal decreased from 115 µg/kg (day 0) to 87 µg/kg (day 10), and hex-2-enal in stored samples (478 µg/kg) significantly decreased compared to those of fresh samples (513 µg/kg) (*p* < 0.05). This is consistent with a previous report in which hex-2-enal content in fresh kiwifruits was approximately twofold higher than that in stored fruits (Huan et al., 2020). In ethylene fruit, hexanal decreased from 158 µg/kg (day 0) to 64 µg/kg (day 10), hex-2-enal significantly decreased from 602 µg/kg (day 0) to 443 µg/kg (day 10) (*p* < 0.05). In 1-MCP fruits, hexanal decreased from 229 µg/kg (day 0) to 77 µg/kg (day 10), and hex-2-enal significantly decreased from 963 µg/kg (day 0) to 772 µg/kg (*p* < 0.05). Despite the decreases in aldehyde concentrations, the rOAV values for hexanal and (E)-2-hexenal remained above 1 during storage, suggesting continued sensory relevance. However, the relative content of aldehydes declined under ethylene treatment, but was partially retained in 1-MCP treated fruit, indicating treatment-dependent preservation of green-like odor characteristics.

Ester concentrations increased during storage in the control and ethylene groups, with corresponding increases in rOAV, indicating enhanced odor activity at later storage stages. The content of seven ester compounds (ethyl acetate, methyl butyrate, methyl isobutyrate, methyl hexanoate, ethyl octanoate, methyl benzoate, and ethyl benzoate) increased after storage. Methyl isobutyrate content of stored control samples (1067 µg/kg) was 97-fold higher than that of fresh control samples (11 µg/kg). Similar storage-associated patterns were observed for methyl butyrate, methyl hexanoate, and ethyl benzoate. In the ethylene treated group, methyl butyrate and ethyl benzoate were not detected in fresh samples but showed 510 µg/kg and 2401 µg/kg, respectively, in stored group. In addition, the methyl isobutyrate content in the stored ethylene-treated samples was 370.9-fold higher than that in the fresh fruits. Notably, methyl butyrate, methyl isobutyrate, and ethyl benzoate reached rOAV > 1 from day 7 in the control group and from day 5 in the ethylene-treated group, indicating that the storage period influenced the ester-associated flavor characteristics. On day 10 of the ethylene treatment, ethyl benzoate showed the maximum rOAV among the identified volatiles. In the 1-MCP treated group, only methyl isobutyrate and methyl benzoate were detected, and ester rOAV remained at < 1 for 10 days, indicating suppressed fruity aroma development. Ethyl acetate is frequently monitored as a representative ester that increases during ripening (Günther et al., 2011). However, its high odor threshold (6200 µg/kg) resulted in consistently negligible rOAV values (< 0.01) in this study, indicating minimal contribution to perceived fruity aroma despite its increased concentration. Accordingly, odor-active benzoates and short-chain esters, particularly ethyl benzoate and methyl isobutyrate, are direct indicators of fruity aroma development in ripe kiwifruit.

These ester responses can be discussed in terms of the LOX pathway-associated aroma biosynthesis and Alcohol Acyltransferase (AcAAT) mediated ester formation during kiwifruit ripening. In a previous study, the expression of AcLOX1, AcLOX5, AcLOX6, and AcAAT was positively correlated with an increase in the total ester content (Wang et al., 2021). Esters in kiwifruit are synthesized by the esterification of acyl-CoA and alcohol in the presence of AcAAT (Zhang et al., 2021a). Accordingly, the suppressed ester accumulation under 1-MCP conditions, relative to the control and ethylene groups, may be consistent with the differential regulation of LOX- and AcAAT-related genes reported during storage.

Alcohol content showed limited treatment effects, with 1-hexanol emerging only at later storage periods (5, 7, and 10 days). The sum of alcohols in stored ethylene treated samples (22 µg/kg) was not significantly different from that in fresh ethylene treated samples (20 µg/kg). In control fruit, the sum of alcohols in stored fruits (24 µg/kg) was 1.6-fold higher than that in fresh samples (15 µg/kg). However, the rOAV values for all identified alcohols remained below 0.1 throughout storage, suggesting a negligible contribution to the aroma characteristics.

Overall, the aroma profiles during storage varied across postharvest treatments, with an increase in ester-driven odor activity in the control and ethylene-treated fruits, whereas this pattern was not observed following 1-MCP application. In the early storage stages, C6 aldehydes (hexanal and hex-2-enal) showed rOAV > 1 across the treatments. Ethyl acetate and terpenes (e.g., α-pinene and β-pinene) showed significant profile level variation across storage and treatments, but their rOAV values indicated minor contributions to odor activity. In contrast, storage-dependent increases in methyl butyrate, methyl isobutyrate, and ethyl benzoate yielded rOAV values above 1 in the control and ethylene-treated fruits. In the 1-MCP treated group, only methyl isobutyrate and methyl benzoate were detected, and ester odorants remained at rOAV < 1 throughout the storage period (days 0 to 10). These findings are consistent with previous reports indicating that aldehydes are associated with green and grassy notes at earlier storage stages, whereas ester formation during ripening contributes to fruity and sweet aroma attributes (Garcia et al., 2012; Huang et al., 2025; Xu et al., 2024). In particular, the increase in short-chain and benzoate esters observed in the control and ethylene-treated fruits suggests their role as late-stage odor-active components associated with fruity or floral notes (Brault et al., 2014).

In conclusion, post-harvest regulation of ethylene activity significantly altered the odor-active volatile profile of Hayward kiwifruit during storage. Twenty-seven volatile compounds, including nine aldehydes, seven esters, four alcohols, five terpenes, one ketone, and one furanone, were identified in the fruits. An odor activity assessment indicated that the early stage profiles were aldehyde-driven, with hexanal, 2-methyl-2-pentenal, and hex-2-enal. During storage, ester odorants (methyl butyrate, methyl isobutyrate, and ethyl benzoate) emerged as key contributors to the control and ethylene-treated fruits, with ethyl benzoate showing the highest odor activity on day 10 in the ethylene-treated group. While ethyl acetate has been widely monitored as a ripening-related ester, its contribution to aroma was limited in this study because of its high odor threshold. Instead, short-chain and benzoate esters have been proposed as relevant aroma-active indicators of kiwifruit ripening. In contrast, 1-MCP inhibited ester accumulation and ester-driven odor activity throughout storage, suggesting suppression of fruity aroma development. Collectively, this study demonstrates how postharvest ethylene regulation alters the major odor-active compounds during the storage of Hayward kiwifruit through the combined evaluation of the relative concentration profiles of volatile compounds and their rOAVs. This quantitative approach supports the translation of analytical measurements into applicable objectives for controlling postharvest aroma during distribution and ripening.

## Supplementary Information

Below is the link to the electronic supplementary material.

Supplementary file1 (DOCX 122 KB)
