# Supplementary material for: Ethylene and 1-methylcyclopropene modulate aroma volatile profiles in kiwifruit (Actinidia spp.) during postharvest storage
Source: Food Sci Biotechnol. 2026 Sep 3;35(13):3621–33. doi: 10.1007/s10068-026-02248-z (PMC13731612; doi:10.1007/s10068-026-02248-z)
Supplement: Supplementary file 1 [file 10068_2026_2248_MOESM1_ESM.docx]

**Supplementary Figure S1.** Total ion chromatogram (TIC) of volatile compounds in Actinidia deliciosa ‘Hayward’ kiwifruit obtained by HS-SPME-GC/MS. Peak numbers correspond to the volatile compounds listed in Table 1.
